# Transcriptome-based identification and expression characterization of RgABCC transporters in *Rehmannia glutinosa*

**DOI:** 10.1371/journal.pone.0253188

**Published:** 2021-06-25

**Authors:** Yan Hui Yang, Chao Jie Wang, Rui Fang Li, Yan Jie Yi, Lei Zeng, Heng Yang, Chang Fu Zhang, Kai Yi Song, Si Jiao Guo

**Affiliations:** College of Bioengineering, Henan University of Technology, Zhengzhou High-technology Zero, Henan Province, 450001, China; Government College University Faisalabad, PAKISTAN

## Abstract

ABCC multidrug resistance-associated proteins (ABCCs/MRPs), a subfamily of ABC transporters, are involved in multiple physiological processes. Although these proteins have been characterized in some plants, limited efforts have been made to address their possible roles in *Rehmannia glutinosa*, a medicinal plant. Here, we scanned *R*. *glutinosa* transcriptome sequences and identified 18 *RgABCC* genes by *in silico* analysis. Sequence alignment revealed that the RgABCCs were closely phylogenetically related and highly conserved with other plant ABCCs/MRPs. Subcellular localization revealed that most of the RgABCCs were deposited in vacuoles and a few in plasma membranes. Tissue-specific expression of the *RgABCCs* indicated significant specific accumulation patterns, implicating their roles in the respective tissues. Differential temporal expression patterns of the *RgABCCs* exhibited their potential roles during root development. Various abiotic stress and hormone treatment experiments indicated that some *RgABCCs* could be transcriptionally regulated in roots. Furthermore, the transcription of several *RgABCCs* in roots was strongly activated by cadmium (Cd), suggesting possible roles under heavy metal stresses. Functional analysis of *RgABCC1* heterologous expression revealed that it may increase the tolerance to Cd in yeast, implying its Cd transport activity. Our study provides a detailed inventory and molecular characterization of the RgABCCs and valuable information for exploring their functions in *R*. *glutinosa*.

## Introduction

ATP-binding cassette (ABC) transporters are one of the largest known superfamilies of membrane transporters in all living organisms [1,2]. ABCC transporters, which belong to one subclass of the ABC transporter superfamily, are well known for their roles as multidrug resistance-associated proteins (MRPs), which are found in all eukaryotic organisms [3–7]. These proteins shuttle substrates as diverse as glutathione conjugates, xenobiotic compounds, intermediate metabolites and hormones across a variety of biological membranes [2,5,8,9]. ABCC transporters from several plants were shown to be responsible for a multitude of functions that included vacuolar sequestration of secondary metabolites [7,10] and heavy metals [2,9], phytohormone transport [5,11], development of plant tissues and response to various stresses [2,5,12].

Each ABCC protein contains at least one highly conserved ATPase domain as an energy source (~200 aa residues long), also referred to as a nucleotide binding domain (NBD) [13]. This ATPase domain comprises Walker A motif and Walker B motif on either end of an ABC signature motif [14]. Corresponding to their sequence similarity, ABCC transporter proteins are classified into two types (i.e., full- and half-molecule) according to their structure and forward orientation [15]. Typically, ABCC proteins, as full-molecule ABC transporters, usually contain two transmembrane domains (TMDs), and two NBDs represented as TMD1-NBD1-TMD2-NBD2 [2,13]. Few half-molecule ABCC transporters are composed of one TMD and one NBD and can be forward (TMD1-NBD1) [16,17]. To date, a number of ABCC transporters have been explored from approximately twenty plant species, such as *Arabidopsis thaliana* (AtABCC1-15) [18], *Oryza sativa* (OsABCC1-17) [19], *Brassica napus* (BnABCC1-48) [20], *Vitis vinifera* (VvABCC1-26) [21], *Triticum aestivum* (TaABCC1-18) [5], *Populus trichocarpa* (PtrABCC1-29) [22], and *Fragaria vesca* (FvABCC1-16) [2] (S1 Table).

*Rehmannia glutinosa*, a species of the *Scrophulariaceae* family, is a perennial herbaceous plant. Its tuberous roots contain a diverse range of pharmacologically active compounds, such as secondary metabolites, possessing various medicinal properties and economic value [23]. Although some ABCC/MRP transporters have been identified or functionally characterized in plants, few attempts have been made to characterize this subfamily from *R*. *glutinosa*. As the genome of *R*. *glutinosa* is still unknown, with the high-efficiency assembly of the transcriptome of this species and the development of bioinformatics tools, the investigation of its *ABCC* genes has become possible [24]. To address the importance of transporters in diverse physiological processes in *R*. *glutinosa*, the molecular structures, phylogeny and conservation of the putative *R*. *glutinosa ABCC* genes (*RgABCC*) were predicted by *in silico* analysis. We analysed RgABCC subcellular localization, investigated their spatio-temporal expression patterns in *R*. *glutinosa* and ascertained their responses to various abiotic stresses as well as plant hormones and Cd stresses. As yeast cadmium factor (YCF1), an ABCC/MRP transporter, was characterized for its possible role in the vacuolar transport of heavy metal sequestration and certain secondary metabolites [5,25], *YCF* defective (*ΔYCF1*) mutants have been utilized as an important resource to address the functional activity of ABCC/MRP orthologues across the kingdom [2,26,27]. Our study also analysed the transport activity of RgABCC1 by YCF1 functional complementation. This laid a basis for further revealing the molecular functions of this subfamily of transporters in *R*. *glutinosa*.

## Materials and methods

### *In silico* analysis

The *R*. *glutinosa* transcriptome data were archived at the NCBI SRA (accession numbers: PRJNA197434) and were assembled and annotated against public data by Li et al. [23]. Based on the transcriptome annotation, putative sequences encoding ABCC proteins of *R*. *glutinosa* were obtained, and their open-reading frames (ORFs) sequences were deduced using the ORFfinder tool [28]. The full-length RgABCC protein sequences were screened for the characteristic features of Walker A, B, and ABCC-MRP-like ATPase domains as defined in the NCBI conserved domains database (CDD) [29] and ScanProsite [30] tools. The RgABCC physicochemical properties were analysed by the ProtParam tool [31]. Topological analysis of the transmembrane helices (TMs) of the RgABCCs was conducted using the TMHMM-2.0 program [32], and their subcellular localizations were predicted using the Plant-mPLoc program [33]. The phylogeny of ABCCs was inferred using the neighbour-joining method implemented in the MEGA v7.0 package, applying 1000 bootstrap replicates and a Poisson correction [34]. The RgABCCs were named according to nomenclature guidelines and the inventory of *A*. *thaliana* ABCC sequences [14]. The domain topology and arrangement of the RgABCCs was predicted using the ScanProsite tool [30,35]. WebLogo3-generated sequence logos were analysed for the presence of representative ABCC domains [36]. Sequence alignment of the ABCC sequences was performed using ClustalX v2.0 software [37].

### Plant material culture

The *R*. *glutinosa* cultivar “Wen 85–5” was cultured in pots in a greenhouse (under a constant temperature of 26°C with a 14-h light/10-h dark cycle and 65% humidity) at the College of Bioengineering, Henan University of Technology. To clone full-ORFs and test tissue-specific expression patterns of *RgABCC* genes, various tissues (including roots, stems, young leaves, functional leaves and old leaves) from five *R*. *glutinosa* plants were sampled at early root expansion stages (i.e., 80 days of cultivation), which is a key point in the transition from fibrous roots to tuberous roots and should be more vigorous in the plant’s gene expression according our previous researches [23,24,38]. To test *RgABCC* temporal expression during root development, roots from five independent plants were collected at the seedling (40 days of cultivation), root elongation (60 days of cultivation), root expansion (early, 80 days, middle, 100 days and late, 120 days of cultivation), and maturity (150 days of cultivation) stages [38]. All the samples were frozen in liquid nitrogen for RNA extraction.

For the various stress treatments, the potted *R*. *glutinosa* seedlings in the above greenhouse were treated at the seedling stage. For the heat treatment, the plants were exposed to 42°C for 24 h. The plants in the salinity and H_2_O_2_ stress treatments were watered with 100 mL of NaCl (150 mM) and H_2_O_2_ (10 mM) solution, respectively. The seedlings not subjected to stress treatment were used as the experimental control. After 24 h of incubation, roots from each plant were collected. For hormone treatments, the potted plants were sprayed with 20 mL of 0.1 mM abscisic acid (ABA), 2 mM ethylene (ETH), and 0.05% gibberellic acid (GA3), and the control plants were sprayed with distilled water. After 24 h of incubation, roots of each of these plants were collected. For Cd stress treatment, the seedlings were irrigated with 100 mL of 100 μM CdCl_2_ solution. The roots of these plants were collected at 0 (i.e., untreated samples as a control), 6, 12, 24, 36 and 48 h. All the samples were stored at −80°C prior to RNA extraction. Three replicates of five plants per pot were used for the above experiments.

### Total RNA isolation and reverse transcription

Total RNA of each tissue sample was extracted using TRIzol™ reagent (Invitrogen, Carlsbad, USA), as recommended by the manufacturer. The RNA concentration was measured spectrophotometrically with a NanoDrop 2000 (Thermo Scientific, Wilmington, DE, USA), and its integrity was evaluated through agarose gel electrophoresis. A 1-μg aliquot of total RNA from each sample was reverse-transcribed into cDNA using HiScript III Reverse Transcriptase (Vazyme, Nanjing, China) according to the manufacturer’s instructions.

### Construction of *RgABCC* vectors

For the *RgABCC* destination vectors, primers for the amplification of the full ORF sequences of the *RgABCCs* extending from the gene’s upstream “ATG” start codon site to the downstream region, including the stop codon, were designed by using Oligo 7.0 software and are shown in S2 Table. The *RgABCC* cDNAs were amplified by polymerase chain reaction (PCR) using PrimeSTAR HS DNA Polymerase. The products were purified with the TaKaRa MiniBEST Agarose Gel DNA Extraction Kit (Takara, Tokyo, Japan) and subcloned into the pMD-18 vector (Takara, Tokyo, Japan), which was then used to transform *E*. *coli*. The constructs were sequenced by the Sanger method (Sangon, Shanghai, China).

For subcellular localization, the ORF cDNAs of the *RgABCCs* were inserted into the pBI121 vector under the control of the CaMV35S promoter and fused with the N-terminus of the GFP gene to generate the CaMV35S:GFP-RgABCC constructs (S1 Fig). For heterologous expression, the cDNA of *RgABCC1* was inserted into the pYES2 vector (Biovector Science Lab, China) under the control of the gal promoter to generate the pYES2-ABCC1 construct (S1 Fig).

### Transient expression analysis

The CaMV35S:GFP-RgABCC constructs and the CaMV35S:GFP empty vector were transformed with *Agrobacterium tumefaciens* GV3101 strains using the freeze-thaw method [39]. For transient expression, the GV3101 strains, which were transformed into the vectors CaMV35S:GFP-RgABCCs and CaMV35S:GFP, were cultured for collection and then infiltrated into onion epidermal cells. The transfected epidermal regions were examined after 48 h of coculture with a fluorescence microscope (FV1000 MPE, Olympus) at an excitation wavelength of 488  nm to visualize GFP fluorescence.

### Quantitative real-time PCR analysis

To determine gene expression, these *RgABCC* primers were designed with Beacon Designer 8.0 software (S3 Table). For quantitative real-time PCR (qRT-PCR) analysis, 2 μg total RNA was reverse-transcribed in a 20 μL reaction containing 5 U M-MLV reverse transcriptase (Takara, Tokyo, Japan) according to the manufacturer’s instructions. Each 25 μL reaction contained 0.2 μM of each primer, 12.5 μL SYBR Premix EX Taq (Takara, Tokyo, Japan) and 100 ng cDNA. The qPCR protocol was as follows: 95°C for 30 s, followed by 36 cycles of 95°C for 10 s, 58–60°C 30 s and 72°C for 30 s to determine the amplicon’s dissociation behaviour. Three biological replicates were included per sample, and three technical replicates were used for each biological replicate. The 2^-ΔΔCT^ method [40] was applied to estimate relative transcript abundances, and the data were normalized to the *RgActin* gene (Genbank ID: EU526396.1) as an internal reference.

### Functional complementation analysis

*YCF1* in the wild-type BY4741 (*MATa*; *his3Δ1; leu2Δ0; met15Δ0; ura3Δ0*) *Saccharomyces cerevisiae* strain was removed by the Cre-LoxP system method [41], and *ΔYCF1* (*MATa; ΔYCF1*::*KanMX2; his3Δ1; leu2Δ0; met15Δ0; ura3Δ0*) mutant strains were generated. Using the lithium acetate/PEG transformation method [42], the *ΔYCF1* mutant cells were transformed with pYES2-RgABCC1 (i.e., pYES2-RgABCC1) and pYES2 empty vectors (named pYES2-RgABCC1-ΔYCF1 and pYES2-ΔYCF1, respectively), and the wild-type BY4741 cells were transformed with pYES2 empty vectors (i.e., pYES2-WT). These cells were precultured in SD-Ura selective liquid medium containing 2% glucose at 30°C for 16 h. The transformed cells were grown overnight to an OD_600_ of 1.5. Aliquots of the cell suspensions were then serially diluted and spotted on solid medium with or without 60 μM CdCl_2_. Colonies were visualized after incubating the plates for 3 days at 30°C. In addition, the strains were grown overnight in liquid medium, and then the cultures were diluted in minimal medium to an OD_600_ of 0.1 in the presence of various concentrations of CdCl_2_ and incubated for an additional 24 h, after which growth was determined by measuring the OD_600_ [43].

### Statistical analyses

All the data were subjected to one-way analysis of variance (ANOVA) using SPSS 22.0 software. Significant differences in the two comparison datasets were analysed by Student’s *t-*test (*p*<0.05 or 0.01). Multiple comparison tests were performed using the least significant difference (LSD) test at *p* < 0.05.

## Results

### Identification and characterization of RgABCC transporters

**Fig 1:**
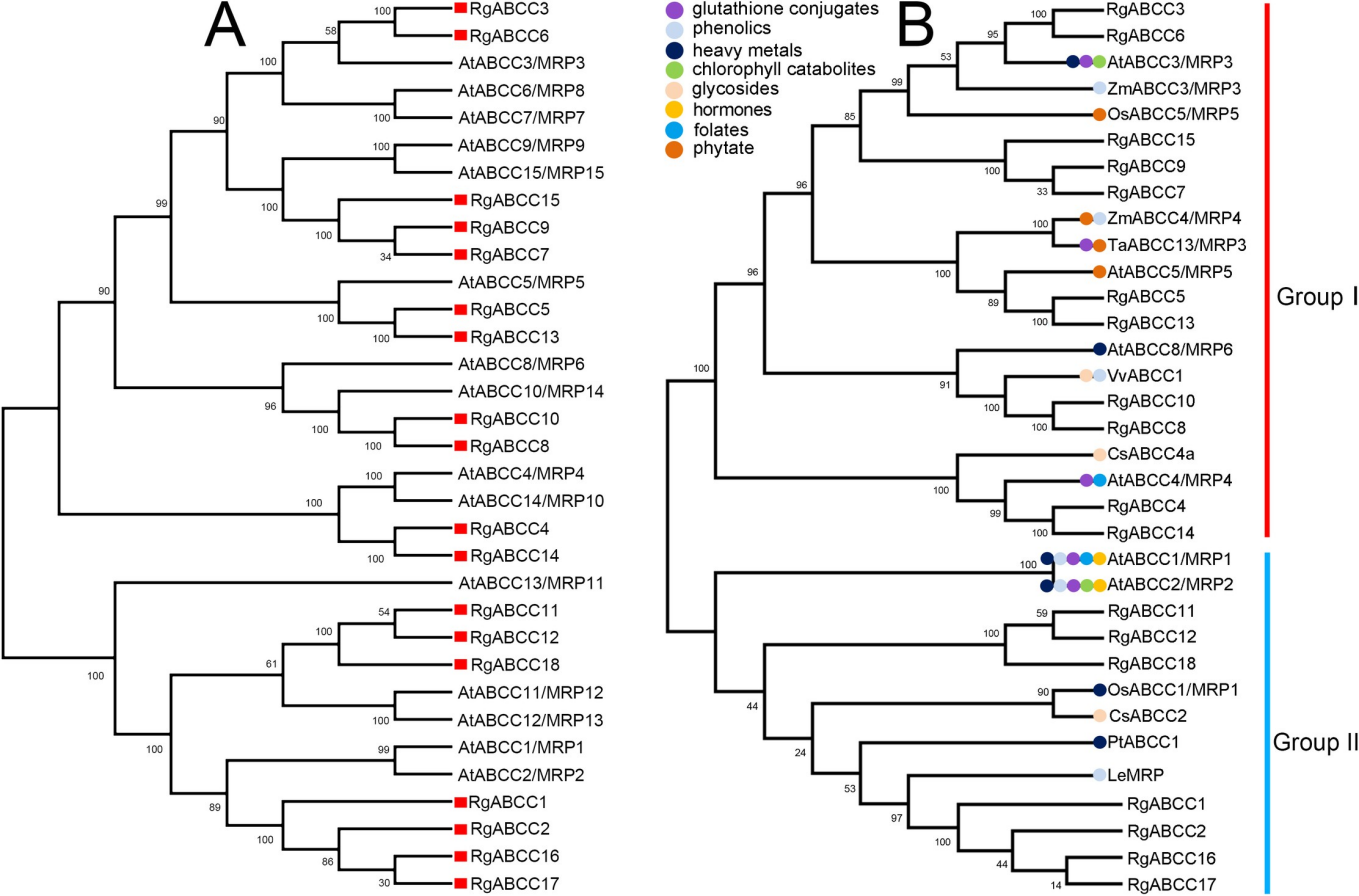
Phylogenetic alignment of plant ABCCs/MRPs. (A) Phylogenetic tree of ABCCs/MRPs from *R*. *glutinosa* and *A*. *thaliana*; (B) Phylogenetic tree of the RgABCCs and functionally characterized ABCCs/MRPs from other plants (Note: Coloured dots indicate the class of transported substrates for the functionally characterized ABCCs/MRPs).

The *R*. *glutinosa* transcriptome was scanned to screen a set of 86 unigenes putatively encoding ABCC transporters (S4 Table). Using the CDD and ScanProsite tools, the full ORF of their translated amino acid sequences (Table 1) were confirmed, and 18 ABCCs from *R*. *glutinosa* were refined. Based on the phylogenetic relationship of ABCCs from *R*. *glutinosa* and *A*. *thaliana* (Fig 1A), we designated RgABCC1 through 18 (Table 1), which were submitted in NCBI Genbank (Accession numbers assigned MW355848 through MW355865). The size of these deduced proteins (Table 1) varied from 844 to 1,622 residues; their predicted molecular masses ranged from 94.97 to 182.32 kDa, their predicted pI values ranged from 5.70 to 8.66 and their TMs ranged from 4 to 17 (Table 1).

**Table 1:** Basic information about the RgABCCs in *R*. *glutinosa*.

Name	Accession number	Length (nt|aa)	Moleculr weight (kDa)	pI	TMD	Topology	Subcelluar location
RgABCC1	MW355848	4869|1622	182.32	6.75	14	(TMD-NBD)_2_	vacuole
RgABCC2	MW355849	4869|1622	182.29	6.75	14	(TMD-NBD)_2_	vacuole
RgABCC3	MW355850	2883|960	106.66	5.90	8	(TMD-NBD)_2_	plasma membrane
RgABCC4	MW355851	4488|1495	167.64	7.49	17	(TMD-NBD)_2_	vacuole
RgABCC5	MW355852	3114|1037	115.31	7.47	5	(TMD-NBD)_2_	plasma membrane
RgABCC6	MW355853	4536|1511	169.34	6.18	15	(TMD-NBD)_2_	plasma membrane
RgABCC7	MW355854	4527|1508	169.36	6.87	12	(TMD-NBD)_2_	vacuole
RgABCC8	MW355855	4440|1479	165.64	7.34	12	(TMD-NBD)_2_	vacuole
RgABCC9	MW355856	2913|970	107.78	5.70	4	(TMD-NBD)_2_	plasma membrane
RgABCC10	MW355857	4440|1479	165.62	7.34	12	(TMD-NBD)_2_	plasma membrane
RgABCC11	MW355858	3774|1257	140.97	8.66	7	(TMD-NBD)_2_	vacuole
RgABCC12	MW355859	3774|1257	140.87	8.55	7	(TMD-NBD)_2_	vacuole
RgABCC13	MW355860	4605|1534	170.43	8.48	14	(TMD-NBD)_2_	plasma membrane
RgABCC14	MW355861	4488|1495	167.60	7.49	17	(TMD-NBD)_2_	vacuole
RgABCC15	MW355862	4527|1508	169.33	6.89	12	(TMD-NBD)_2_	vacuole
RgABCC16	MW355863	3774|1257	140.40	6.68	8	(TMD-NBD)_2_	vacuole
RgABCC17	MW355864	2535|844	94.97	5.98	5	TMD-NBD	vacuole
RgABCC18	MW355865	2619|872	98.09	6.82	5	TMD-NBD	vacuole

To assign their potential functional roles, a phylogenetic tree was constructed that compared the sequences of the RgABCCs with 16 functionally characterized ABCC/MRPs from other plants (Fig 1B and S5 Table). Phylogenetic analysis showed that these ABCCs/MRPs primarily formed a cluster with two separate clades (Group I and Group II). In Group I, RgABCC3 and RgABCC6 were clustered closely together with AtABCC3/MRP3, ZmABCC3/MRP3 and OsABCC3/MRP3; RgABCC7, RgABCC9 and RgABCC15 were clustered together with AtABCC9/MRP9, AtABCC15/MRP15, OsABCC6/MRP6 and OsABCC7/MRP7; RgABCC8 and RgABCC10 were closely related to AtABCC5/AtMRP5; in addition, RgABCC8 and RgABCC10 were closely related to VvABCC1, while RgABCC4 and RgABCC14 exhibited a close relationship to AtABCC4/MRP4 and CsABCC4a. In group II, seven RgABCCs (e.g., RgABCC1, RgABCC2, RgABCC11, RgABCC12, RgABCC16, RgABCC17 and RgABCC18) were found to be closely associated with AtABCC1/MRP1 and AtABCC2/MRP2, and four other known ABCCs (OsABCC1/MRP1, CsABCC2, PtABCC1 and LeMRP) and five RgABCCs (e.g., RgABCC1, RgABCC2, RgABCC16, RgABCC17 and RgABCC18) were found to be closely clustered with PtABCC1.

**Fig 2:**
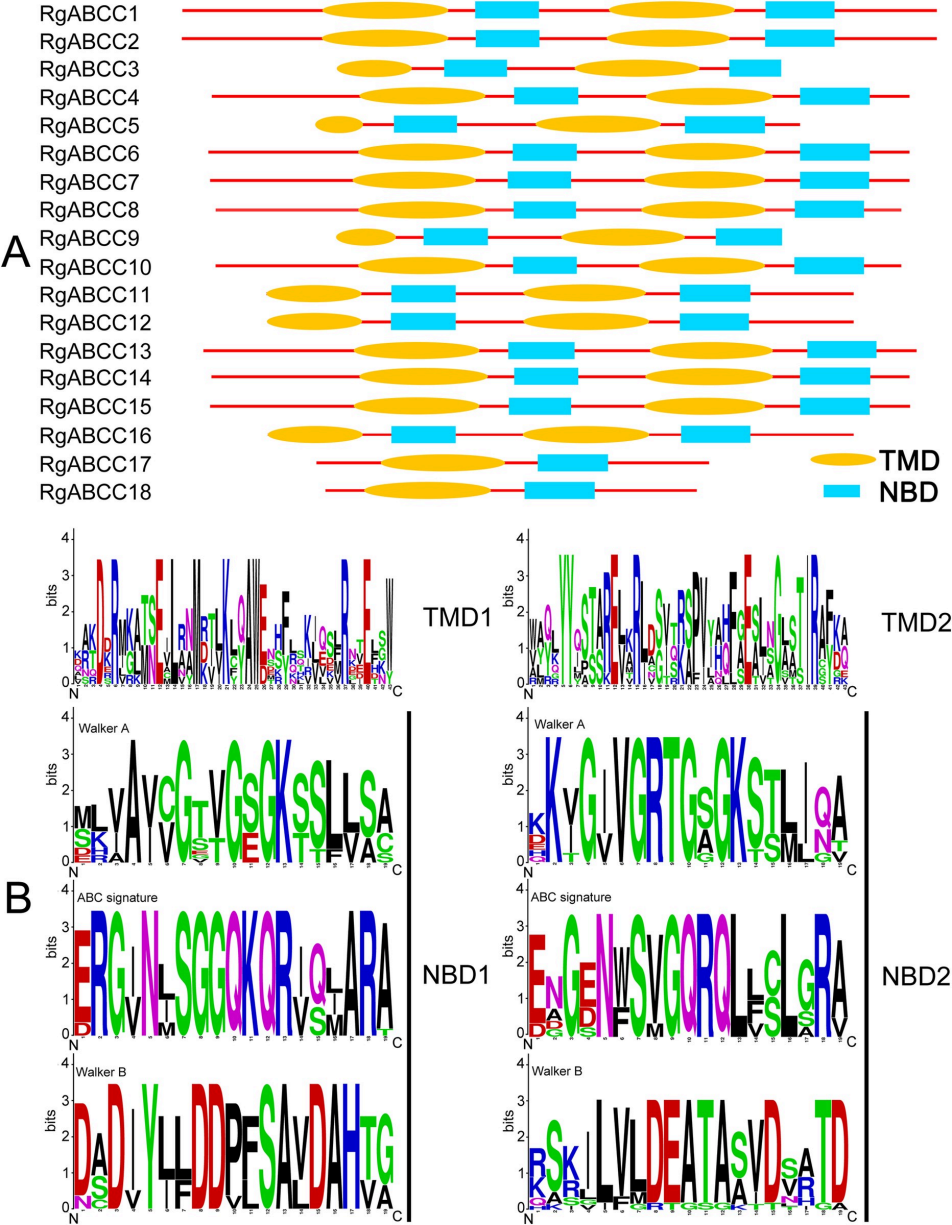
Conserved domain analysis of the RgABCCs. (A) Schematic diagram of the domain arrangement in different RgABCCs; (B) sequence logo of amino acids conserved in different domains (Note: The Y-bits represent conservation of amino acids at that position (height)).

Conserved domain analysis showed that the specific signatures of the RgABCCs were present in 16 full-molecule (TMD-NBD-TMD-NBD) and 2 half-molecule (TMD-NBD) members (Fig 2A and Table 1). Sequence logos strongly suggested more conserved amino acids in the RgABCC domains (Fig 2B). All of the RgABCCs had a mainly conserved NBD1 represented by GTVGSGK amino acids of the Walker A motif, SGGQKQR of the ABC signature motif and IYLLD of the Walker B motif. Similarly, the NBD2 domains with the Walker A (GRTGSGK), the ABC signature (SVGQRQL) and the Walker B (ILVLD) motifs were also highly conserved and present among the full-molecule RgABCCs (Fig 2B). Based on homology analysis among the RgABCCs, RgABCC1 showed the highest identity (97.94%) with RgABCC2 (S6 Table). In contrast, the highest divergence was observed between RgABCC9 and RgABCC17, with 10.43% identity. To assess the conservation of the RgABCCs across various species, we collected 16 functionally characterized ABCC/MRPs from other plants (S7 Table). When a cross-species comparison was performed, the highest percentage identity of 81.18% was observed for RgABCC1 and PtrABCC1 of *P*. *trichocarpa*. Similar to *A*. *thaliana*, the highest percentage identity of 77.33% was observed for RgABCC1 or RgABCC2 with AtABCC2/MRP2.

### Subcellular localization of the RgABCCs

**Fig 3:**
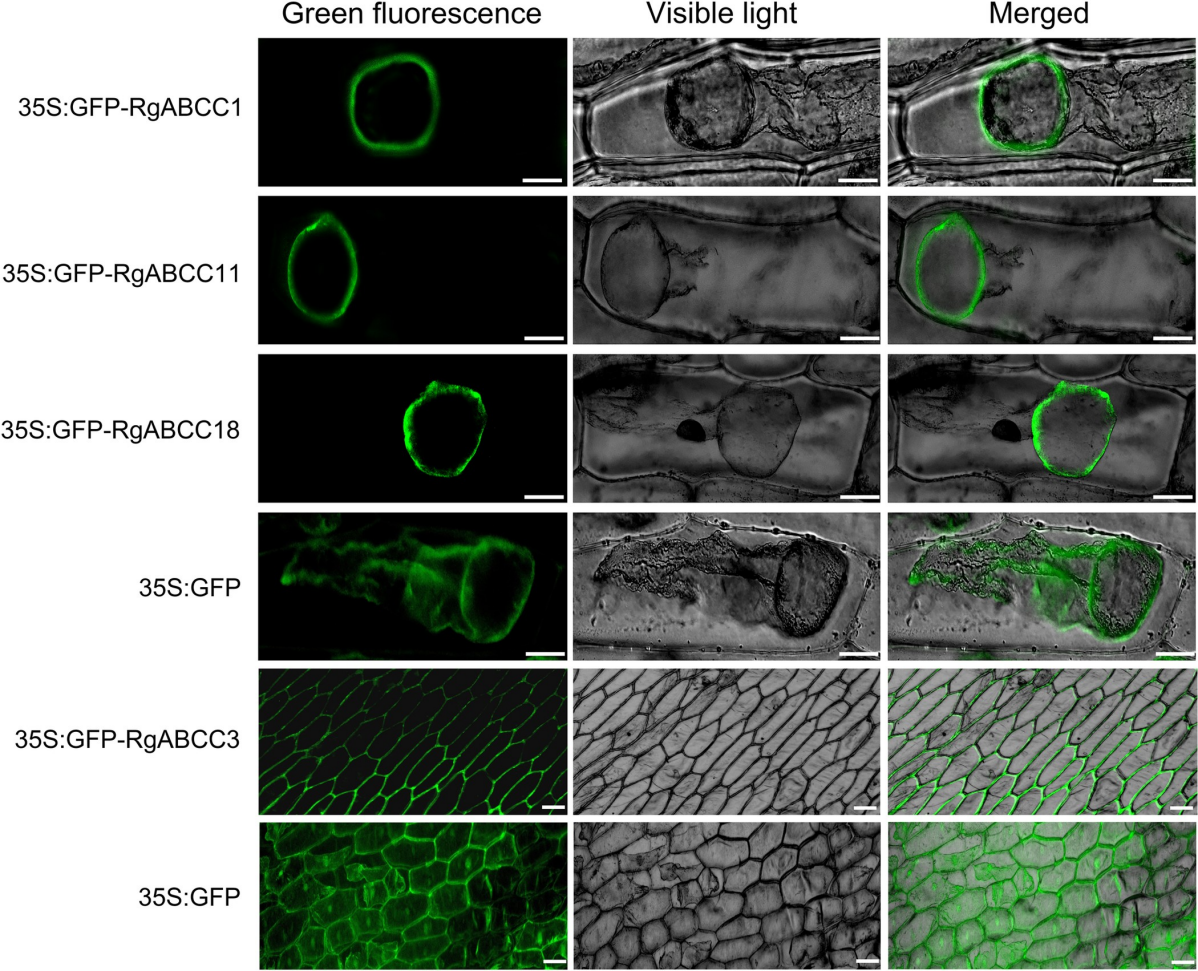
Subcellular localization of RgABCCs. The expression of CaMV35S:GFP-RgABCC fusion proteins in onion epidermal cells (Bars = 320 μm).

Based on *in silico* analysis, of these RgABCCs, 12 were predicted to localize to the vacuole and 6 to the plasma membrane (Table 1). Four of them (RgABCC1, RgABCC3, RgABCC11 and RgABCC18) were selected for experimental determination of their subcellular localization. The recombinant proteins from the CaMV35S:GFP-RgABCC constructs were transiently expressed in onion epidermis. Green fluorescence from the fusion proteins of CaMV35S:GFP-RgABCC constructs was mainly observed in the vacuoles (RgABCC1, RgABCC11 and RgABCC18) and plasma membranes (RgABCC3) (Fig 3), while the expression of the control CaMV35S:GFP was detected in the plasma membrane, cytoplasm, nucleus or other cell organelles (Fig 3). The results confirmed the predicted subcellular localizations of these RgABCCs.

### Spatio-temporal expression patterns of the *RgABCCs* in *R*. *glutinosa*

**Fig 4:**
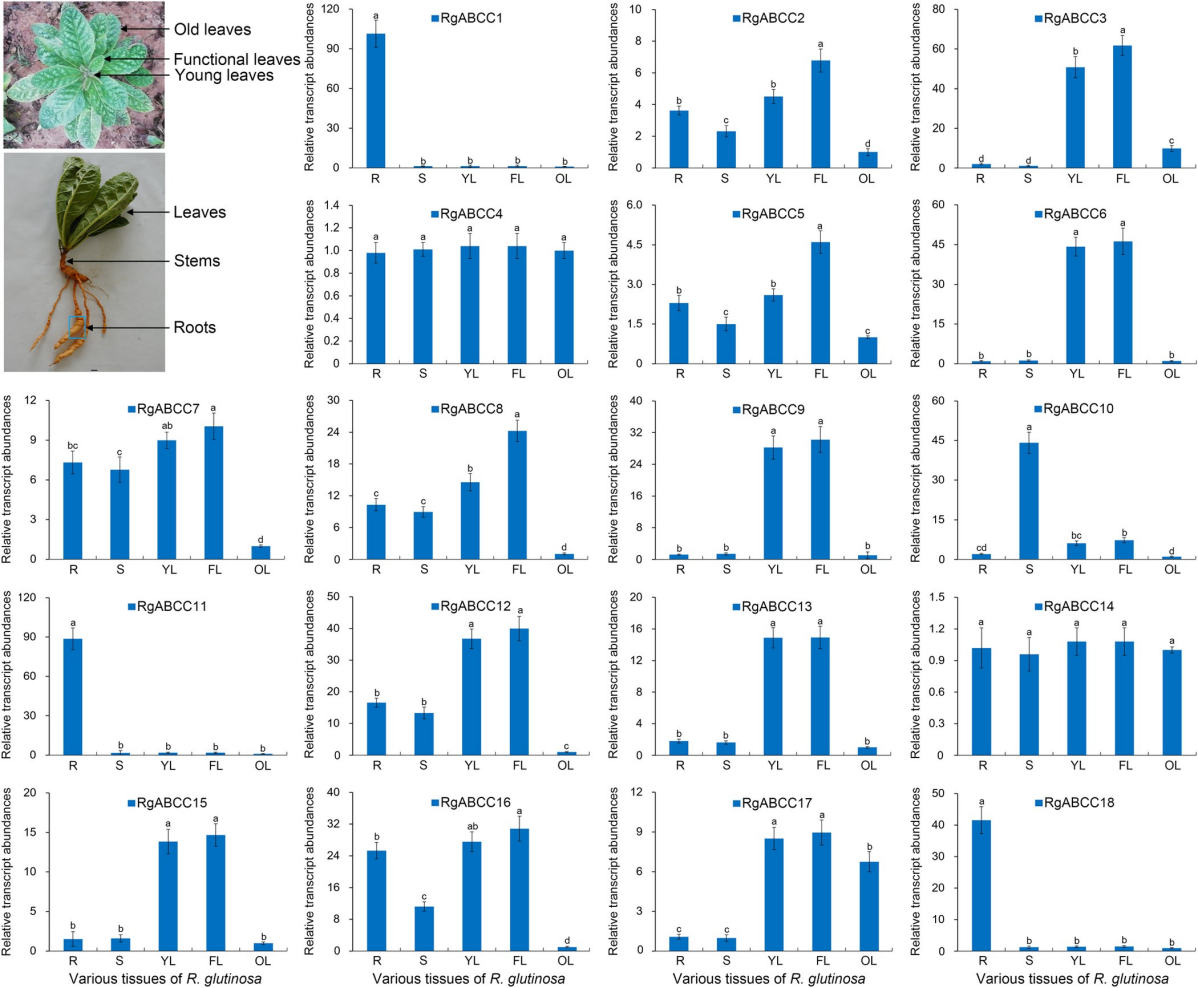
Transcript profiles of the *RgABCCs* in various tissues. Note: “R, S, YL, FL and OL” represent “roots, stems, young, functional and old leaves, respectively; errors bars are standard deviation (SD), the same below.

To gain insight into the expression patterns of the *RgABCCs* in various tissues of *R*. *glutinosa*, qRT-PCR analysis was performed (Fig 4 and S8 Table). Overall, among the *RgABCCs*, *RgABCC4* and *RgABCC14* were ubiquitously expressed in all tissues, exhibiting no significant difference in transcript abundances; the remaining 16 *RgABCCs* exhibited differential transcription responses in various tissues. For example, the transcript abundances of *RgABCC12* in the roots, stems, young leaves and functional leaves were 16.56-, 13.34-, 36.72- and 39.92-fold higher, respectively, than those in the old leaves. Moreover, 15 of the remaining 16 *RgABCCs* (except *RgABCC18*) exhibited extremely low transcript abundances in the old leaves, i.e., the expression of 6 *RgABCCs* (i.e., *RgABCC2*, *RgABCC5*, *RgABCC7*, *RgABCC8*, *RgABCC12 and RgABCC16*) exhibited similar patterns in these tissues, which were the highest in functional leaves, followed by young leaves, roots and stems, with the lowest abundances in old leaves, whereas the 5 *RgABCCs* including *RgABCC3*, *RgABCC6*, *RgABCC9*, *RgABCC13* and *RgABCC15* exhibited the highest transcript abundances only in young leaves and functional leaves. For example, the abundances of *RgABCC12* in the functional and young leaves, roots and stems were 16.56-, 13.34-, 36.72- and 39.92-fold those in the old leaves, whereas the abundances of *RgABCC6* in the young leaves and functional leaves were 45.11- and 47.14-fold those in the roots. In addition, the transcript abundance of *RgABCC17* was significantly higher in these leaves than in roots and stems (Fig 4 and S8 Table), and the abundance of *RgABCC10* was the highest in the stems. Surprisingly, compared with those in other tissues, the transcript abundances in the roots were significantly highest for *RgABCC1* (approximately 70-fold) followed by *RgABCC11* (50-fold) and *RgABCC18* (30-fold) (Fig 4). However, only *RgABCC10* abundance was the highest in the stems compared with other tissues, exhibiting levels 21.85-, 7.27-, 6.07- and 44.13-fold in roots, stems, young leaves and functional leaves, respectively. The differential expression patterns of these *RgABCCs* suggested that they had diverse functions in *R*. *glutinosa*.

**Fig 5:**
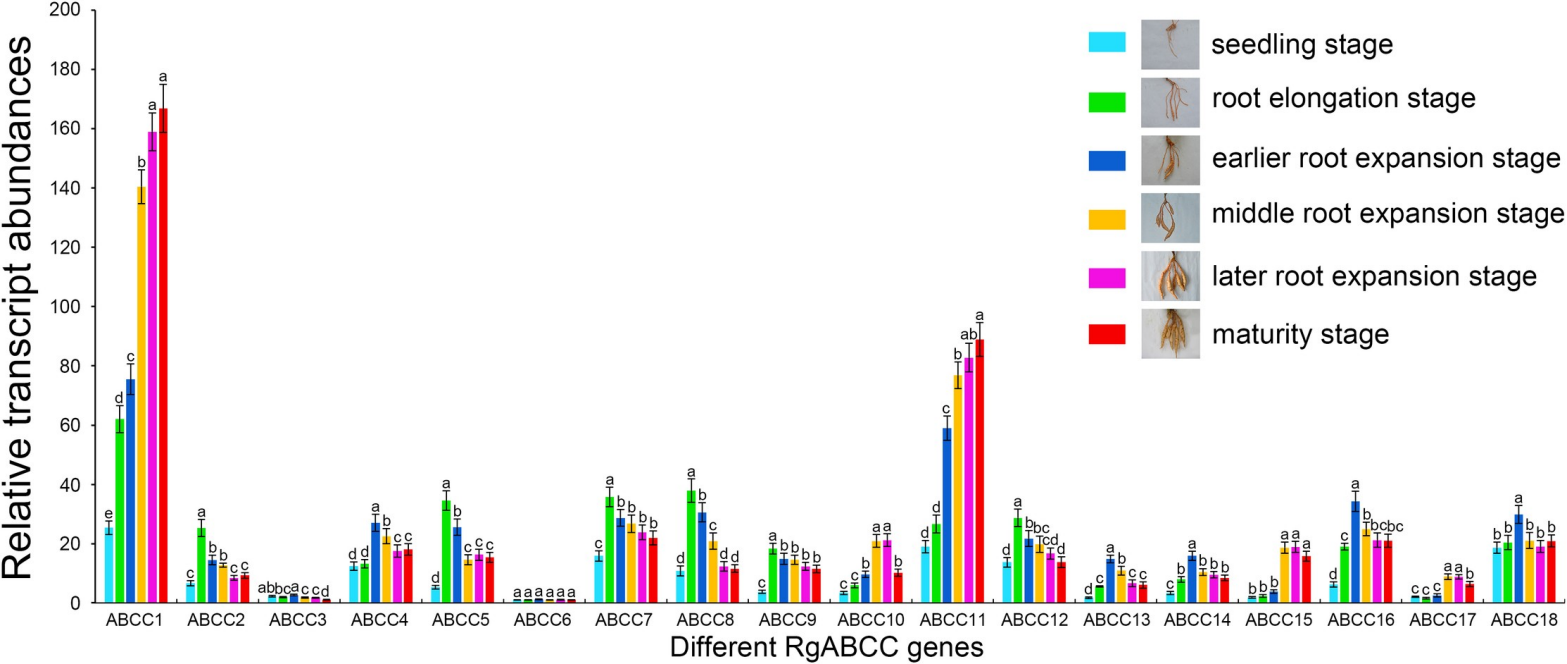
Transcript profiles of the *RgABCCs* at various root development stages.

Moreover, the expression patterns of the *RgABCCs* during root development of *R*. *glutinosa* were assessed by qRT-PCR analysis. As shown in Fig 5 and S9 Table, compared with the seedling stage, the transcript abundances of most *RgABCCs* (except *RgABCC3* and *RgABCC6*) exhibited significantly differential increases at the other five stages. We found that the transcript abundances of *RgABCC1* and *RgABCC11* increased with the prolongation of cultivation days (especially at maturity); six *RgABCCs* (i.e., *RgABCC2*, *RgABCC5*, *RgABCC7*, *RgABCC8*, *RgABCC9* and *RgABCC12*) showed the highest transcript abundances at the root elongation stage, five (*RgABCC4*, *RgABCC13*, *RgABCC14*, *RgABCC16* and *RgABCC18*) exhibited the highest transcript abundances at the earlier root expansion stage, and three (*RgABCC10*, *RgABCC15* and *RgABCC17*) showed the highest abundances at both the middle and later root expansion stages. For example, the transcript abundance of *RgABCC8* at the root elongation stage was 3.49-fold that at the seedling stage, the abundance of *RgABCC14* at the earlier root expansion stage was 4.73-fold that at the seedling stage, and the abundances of *RgABCC15* at both the middle and later root expansion stages were 7.94- and 8.05-fold that at the root elongation stage, respectively. Furthermore, among the *RgABCCs*, the transcript abundances of *RgABCC1* and *RgABCC11* were prominently higher than those of other *RgABCCs* during root development, whereas those of *RgABCC3* and *RgABCC6* were much lower than those of other *RgABCCs*. For example, the transcript abundances of *RgABCC1* and *RgABCC11* were 152.86- and 79.58-fold that of *RgABCC6* at the later root expansion stage, respectively. The results suggested that the transcription of the *RgABCCs* responded differently during the development of *R*. *glutinosa* roots.

### Transcript profiles of the *RgABCCs* under various stresses

**Fig 6:**
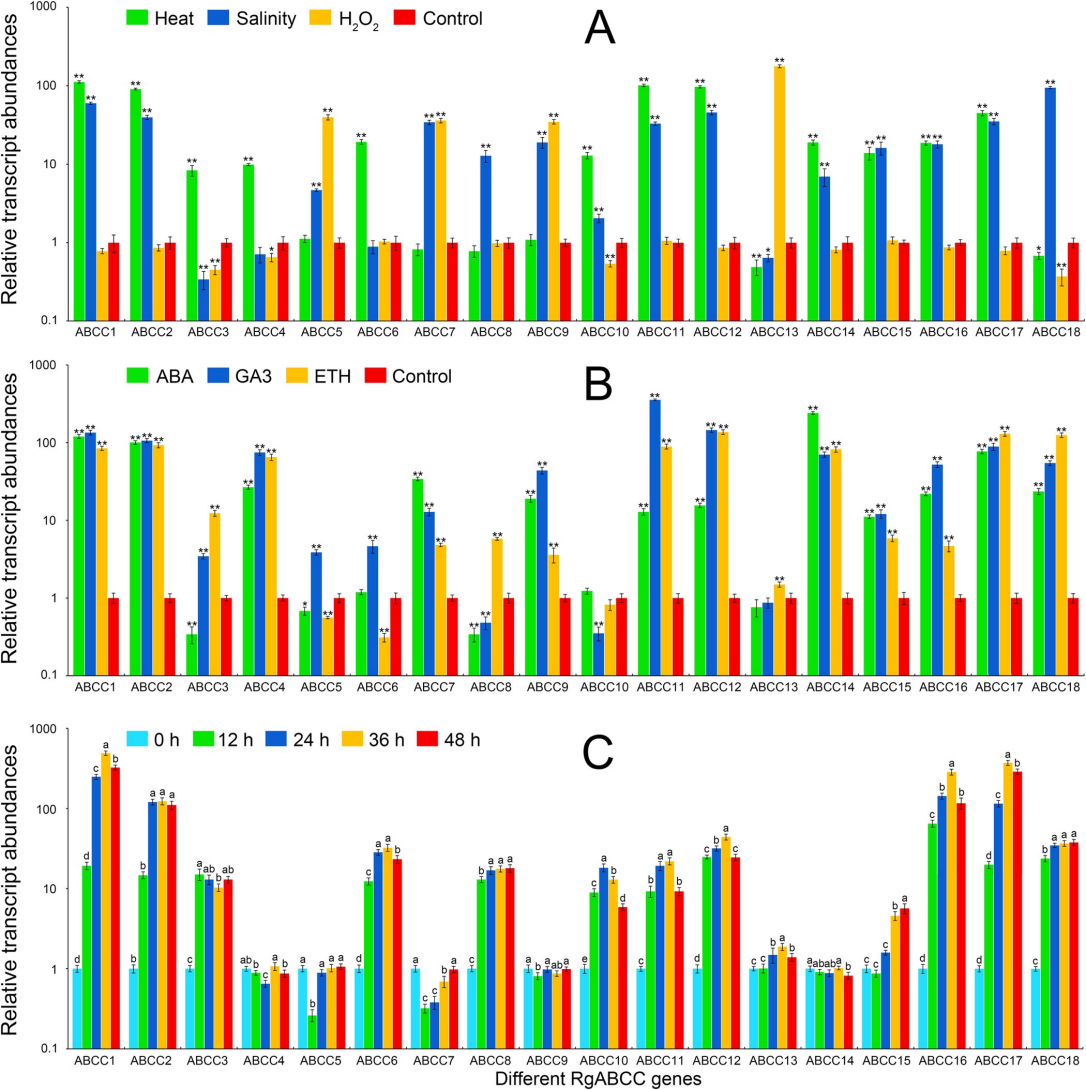
Transcript profiles of the *RgABCCs* under various conditions. (A) three abiotic stresses; (B) three hormone treatments; (C) CdCl_2_ stress.

To investigate the roles of the *RgABCCs* in the adaptation of plants to various abiotic stresses, qRT-PCR was performed to examine the responses of these genes to heat, salinity and H_2_O_2_ stresses in *R*. *glutinosa* roots. The transcript profiles of these genes exhibited differences under various stresses. As shown in Fig 6 and S10 Table, most *RgABCC* transcript profiles were changed under these stresses. Compared with the control, 14 of the *RgABCCs* in the roots, except for *RgABCC7*, *RgABCC8*, *RgABCC13* and *RgABCC18*, were upregulated under heat stress; moreover, the abundance of *RgABCC1* was the highest, peaking at 111.67-fold relative to the control. The increased transcript abundances of *RgABCC5* and *RgABCC9* were not obviously different relative to the control, whereas the abundances of *RgABCC7*, *RgABCC8*, *RgABCC13* and *RgABCC18* were decreased. Under salinity stress, 14 *RgABCCs*, except for *RgABCC3*, *RgABCC4*, *RgABCC6* and *RgABCC13*, were upregulated. In particular, *RgABCC18* had the highest abundance, peaking at 94.56-fold relative to the control, whereas *RgABCC3* was downregulated 0.34-fold. However, under H_2_O_2_ stress, a few of the *RgABCCs* (i.e., *RgABCC5*, *RgABCC7*, *RgABCC9* and *RgABCC13*) were highly upregulated, whereas other *RgABCCs* were downregulated or showed no response. For example, *RgABCC13* had the highest abundance among the genes and peaked at 173.71-fold relative to the control, whereas *RgABCC18* had the lowest abundance, 0.37-fold relative to the control. Obviously, the homologous *RgABCCs* exhibited different transcript profiles, implying a functional divergence of the homologous genes to these abiotic stress responses.

Moreover, the response of the *RgABCCs* to plant hormones (ABA, ETH and GA3) was also investigated in *R*. *glutinosa* roots (Fig 6 and S10 Table). When exposed to ABA conditions, 14 *RgABCCs* were upregulated, whereas only four *RgABCCs* (i.e., *RgABCC3*, *RgABCC5*, *RgABCC8* and *RgABCC13*) were downregulated; in particular, *RgABCC14* had the highest abundance and peaked at 240.93-fold relative to the control, whereas *RgABCC8* was downregulated 0.34-fold. After GA3 treatment, 15 of the *RgABCCs* were upregulated (except for *RgABCC8*, *RgABCC10* and *RgABCC13*); in particular, *RgABCC11* had the highest abundance and peaked at 355.71-fold, whereas *RgABCC10* was significantly downregulated 0.35-fold by GA3 induction (Fig 6). After ETH induction, 15 *RgABCCs* were upregulated, except for *RgABCC5*, *RgABCC6* and *RgABCC10*; in particular, *RgABCC12*, *RgABCC17*, and *RgABCC18* had higher abundances, peaking at 136.89-, 129.81- and 124.89-fold relative to the control.

To investigate the potential roles of the *RgABCCs* in the heavy metal stress response, these gene transcription patterns were assessed in *R*. *glutinosa* roots exposed to Cd stress at different time points. After Cd treatment, most of the *RgABCCs* exhibited different transcript abundances in response to Cd stress (Fig 6 and S10 Table). For example, *RgABCC1* and *RgABCC17* were significantly upregulated during the Cd treatment, peaking at 488.93- and 369.81-fold, respectively, at the 36-h point. *RgABCC2* and *RgABCC16* were also highly upregulated in response to the Cd treatment. In contrast, six *RgABCCs*, including *RgABCC4*, *RgABCC5*, *RgABCC7*, *RgABCC9*, *RgABCC13* and *RgABCC14*, were mostly downregulated or showed no significant differences in the response to the Cd treatment.

### Functional complementation analysis of RgABCC1

**Fig 7:**
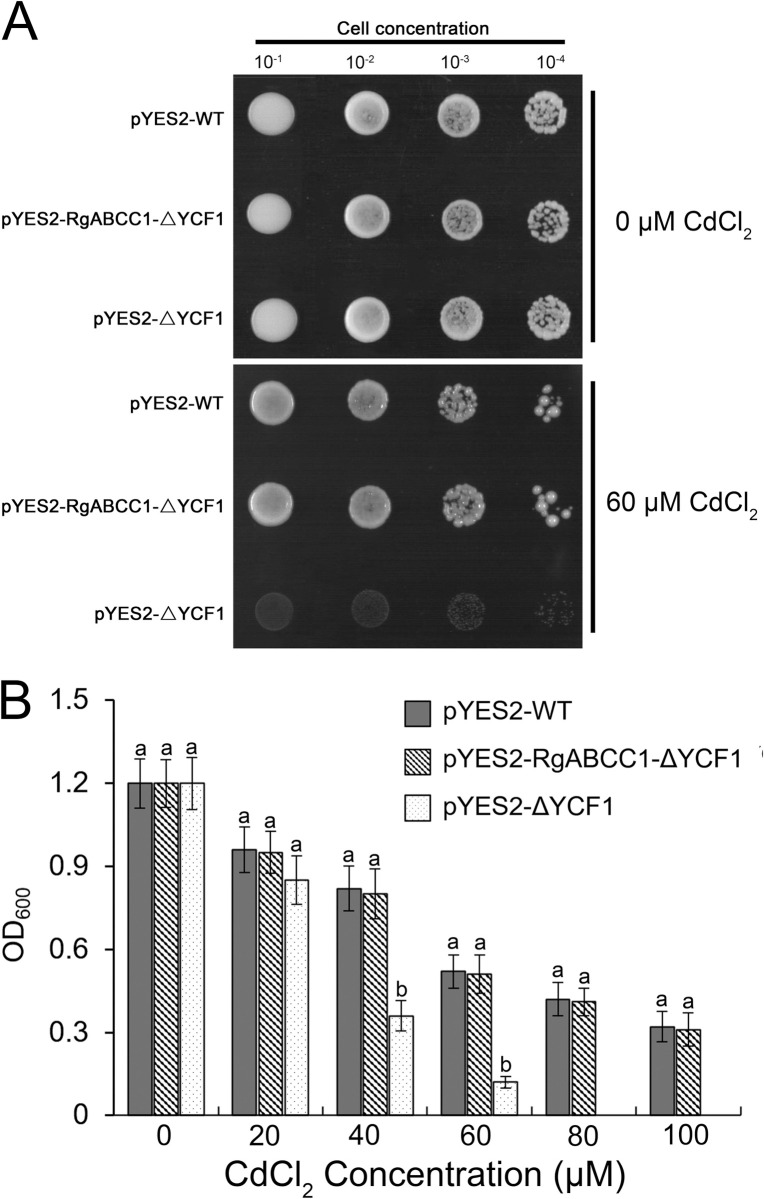
Cd tolerance analysis for *RgABCC1* heterologous expression in the *ΔYCF1* yeast mutant. (A) Drop assay of the strain expressing *RgABCC1* on medium with 0 or 60 μM CdCl_2_; (B) Growth assay of the strain expressing *RgABCC1* under CdCl_2_ stress at various concentrations.

To identify whether RgABCC1 was capable of Cd transport in cells, the *ΔYCF1* mutant cells were transformed with constructs containing an empty vector pYES2 and pYES2-RgABCC1, which were named pYES2-RgABCC1-ΔYCF1 and pYES2-ΔYCF1, respectively; and the wild-type cells were transformed with an empty vector pYES2, which were named pYES2-WT. The yeast cells were cultured on SD-Ura solid medium lacking or containing 60 μM CdCl_2_ (Fig 7A). Under non-Cd condition, the growth phenotypes of the cells were not different; however, under Cd stress, the growth of the pYES2-RgABCC1-ΔYCF1 cells was similar to that of pYES2-WT, whereas the pYES2-ΔYCF1 cells grew much worse than those of pYES2-WT. Moreover, we measured the density phenotypes of the three cell types cultured in liquid medium lacking or containing CdCl_2_ (Fig 7B and S11 Table). In the medium lacking CdCl_2_, the growth of the PYES2-RgABCC1-ΔYCF1 and pYES2-ΔYCF1 cells was similar to that of pYES2-WT cells. In the presence of 20–100 μM CdCl_2_, the density of pYES2-ΔYCF1 cells decreased in a concentration-dependent manner. However, the densities of PYES2-RgABCC1-ΔYCF1 and pYES2-WT cells were not significantly different, at approximately 0.3 at OD_600_ in the presence of 100 μM CdCl_2_ (Fig 7B). These results indicated that the expression of *RgABCC1* in *ΔYCF1* mutants conferred tolerance to CdCl_2_.

## Discussion

### Identification and characterization of the RgABCCs

ABCC/MRPs, a subfamily of the larger ABC gene family, transport a wide range of molecules involved in plant growth and development, heavy metal detoxification, biotic and abiotic stresses, and the accumulation of endogenous active compounds and other physiological processes [1,14,44,45]. A number of ABCCs/MRPs have been well documented in plant species [2,5,10]. Our study identified a set of putative *RgABCCs* based on *R*. *glutinosa* transcriptome data. Phylogenetic analysis revealed that the RgABCCs had close relations to these functionally characterized homologous proteins from other plants (Fig 1B). To date, these functionally characterized ABCCs have been found to transport specific or multiple substrates [1,44–46]. For example, in the phylogenetic tree, OsABCC1/MRP1 and PtABCC1 of group II were prominently responsible for heavy metal transport [9,46], and two CsABCCs (i.e., CsABCC4a of Group I and CsABCC2 of Group II) were responsible for the transport of natural crocins [10], whereas AtABCC1/MRP1, AtABCC2/MRP2 and AtABCC3/MRP3 may be responsible for the transport of diverse substrates (such as heavy metals, glutathione conjugates and some secondary metabolites) [11,47–49]. Thus, we speculated that the RgABCCs, regardless of whether they were classified into group I or group II, could be responsible for the transport of specific or multiple substrates involved in various biological processes of *R*. *glutinosa*.

The structure of a typical ABCC protein is that of a full-molecule ABC transporter, which includes four core domains, i.e., two NBDs and two TMDs [14,50]. Among the RgABCCs, 16 possessed these typical core domains and were considered full-molecule ABCC transporters like most other plant ABCCs [2,5]. However, the functional unit of a few ABCC proteins showed half-molecule transport, and the configuration of the TMD-NBD has been reported in several plants [16,17,51]. Our study found that two RgABCCs (i.e., RgABCC17 and RgABCC18) showed half-molecule characteristics and had a topological pattern similar to that reported in the ABCC proteins of soybean and tomato [17,51]. Conservation analysis revealed that the RgABCCs were highly homologous to other plant ABCCs, specifically retaining the set of the architectural NBD domains, such as the Walker A motifs, the Walker B motifs and the ABC signatures [13,52,53]. Therefore, this implied that the potential functions of the RgABCCs are similar to those of the ABCC/MRPs of other plant species [17,51].

In plants, most ABCC/MRPs characterized to date are vacuole-localized proteins that mediate detoxification, sequestration and accumulation of endogenous or exogenous secondary metabolites and toxins [5,10,47], and a few of them have been reported to reside on the plasma membrane, controlling the plasma membrane anion channels of guard cells [12,13]. Here, subcellular localization prediction of the RgABCCs revealed that the majority of proteins were located in the vacuoles, while a few of proteins were located in the plasma membrane, in agreement with the homologous proteins in rice, *A*. *thaliana* and maize [5,7,12,13,16]. Transient expression analysis verified that three RgABCCs were localized to the vacuoles, while RgABCC3 was localized to the plasma membrane. Thus, subcellular localization analysis revealed that the RgABCCs might have potential transmembrane transport functions like other documented plant ABCCs [2,5,47,53]. *R*. *glutinosa* as a traditionally medicinal plant can produce extremely diverse specialized metabolites (such as phenolics, phenylethanoid glycosides and rehmanniosides) [23,24,38], which is mainly synthesized in the cytosol and must be transported to the vacuole by one or more vacuolar transporters [7,10,47]. As previous researches revealed that most plant ABCCs can transport numerous phenolics and glycosylated metabolites into the vacuoles [2,7,47,54], we thought most vacuole-localized RgABCCs might be related to the transport of diverse specialized metabolites in *R*. *glutinosa*.

### Different spatio-temporal expression patterns of the *RgABCCs* reflected functional diversification

As preferential gene expression patterns suggest specificities for certain tissues/organs [2,5,54], investigation of the expression patterns in specific tissues/organs provides molecular clues for the roles of the *RgABCCs* and helps to explore their functions in *R*. *glutinosa*. Our data indicated that *RgABCC1*, *RgABCC11* and *RgABCC18* exhibited significantly higher transcript levels in the roots, implying potential transport activities in the root growth and development process, whereas *RgABCC3* and *RgABCC6* are preferentially expressed in the young and functional leaves, implying their functional roles in leaf development. Specifically, *RgABCC10* had higher transcription in the stems than other *RgABCCs*, and its protein sequence exhibited a closer phylogenetic relationship to VvABCC1, an anthocyanidin glucoside transporter [54], implying a similar specific function in *R*. *glutinosa* stems. However, *RgABCC4* and *RgABCC14* transcripts were found to be equally abundant in various tissues, and both were present in the same subcluster of Group I from the phylogenetic tree as AtABCC4/MRP4 and CsABCC4a, which participate in the vacuolar transport of glutathione-conjugates, glycosides and folates [10,12,18], implying such potential transport activities for RgABCC4 and RgABCC14.

Due to the presence of multiple genes for the *RgABCCs* and their different transcript abundances during root development, we expected to uncover some vital functional members. Our data indicated that the transcript accumulation of *RgABCC1* and *RgABCC11*, which were predicted to localize in the vacuoles, exhibited obviously higher expression during the entire root development process, and they formed a cluster (Group II) with several functional ABCC/MRPs, including AtABCC1/MRP1, AtABCC2/MRP2, CsABCC2 and LeMRP, by phylogenetic analysis. In *A*. *thaliana*, AtABCC1/MRP1 and AtABCC2//MRP2 are critical for vacuole transport and the accumulation of endogenous secondary metabolites, including anthocyanins, flavonoids and folates [7,55]. In *Crocus sativus*, CsABCC2 mediates the vacuolar accumulation of crocins [10]. In *Lithospermum erythrorhizon*, LeMRP was identified as being responsible for shikonin transport and accumulation during the hairy root development process [56]. As *R*. *glutinosa* roots are active parts for the accumulation of important medicinal secondary metabolites (e.g., phenolics and glycosides), higher expression of *RgABCC1* and *RgABCC11* in roots might be related to the transport and accumulation of some active ingredients, such as phenolics or glycosides, although further functional studies are required for verification. Moreover, *RgABCC10*, *RgABCC15* and *RgABCC17* exhibited preferential expression in the middle and later root expansion stages, implying their potential roles in root trait development. However, *RgABCC3* and *RgABCC6* exhibited extremely low transcript abundances during root development, whereas both genes were prominently expressed in young leaves and functional leaves, and *RgABCC3* and *RgABCC6* were closely related to AtABCC3/MRP3, which is involved in chlorophyll catabolite transport [47], and ZmABCC3, which is involved in anthocyanin transport [57]. Leaves are an important organ for the biosynthesis and accumulation of transitory nutrients and secondary metabolites that contribute to root expansion [58]. Thus, we speculated that the higher transcription of *RgABCC3* and *RgABCC6* in leaves might be involved in the transport of some nutrients and secondary metabolites to guarantee *R*. *glutinosa* tuberous root expansion. However, the *RgABCCs* exhibited different temporal expression patterns in *R*. *glutinosa* roots, demonstrating the functional specificities for these respective stages.

### The *RgABCCs* involved in abiotic stress responses

Previous studies revealed that some ABCCs are involved in the molecular regulation of abiotic stresses [59–61]. In strawberry, most of the FvABCC subfamily expression was positively responsive to heat and salt treatment [2]. Our data indicated that the transcription of most *RgABCC*s was positively induced by heat and salt stresses, implying that the *RgABCCs* could be involved in the defence against heat and salt stresses. In particular, the transcription of *RgABCC1* was prominently induced by heat treatment, implying that the high temperature stress could promote many secondary metabolisms of *R*. *glutinosa*, leading to produce more secondary metabolites and enhance the RgABCC transport activity; similarly, the transcription of *RgABCC18* was highly induced by salinity treatment, implying its potential function in response to salinity stress. However, transcription was activated by H_2_O_2_ treatment (as an oxidant stress) in only four *RgABCCs*, especially *RgABCC13*, which was prominently activated by the stress. In wheat, *TaABCC13* was significantly induced by H_2_O_2_ treatment [5], and RgABCC13 was closely clustered with TaABCC13, implying its importance in the antioxidant stress response in *R*. *glutinosa*.

Some ABCCs are involved in plant hormone transport and regulation [2,5,62]. Here, most *RgABCCs* were highly induced by ABA treatment; the result was the same for *AtABCC13/MRP11* of *A*. *thaliana* [62] and *FvABCCs* of strawberry [2]. It was reported that in *A*. *thaliana*, *AtABCC1/MRP1* and *AtABCC2/MRP2* responded to ABA treatment and were critical for vacuolar sequestration of abscisic acid glucosyl ester [11]. The *RgABCCs* (*RgABCC11*, *RgABCC12* and *RgABCC18*) were also highly induced by GA3 treatment; this finding is supported by previous transcription patterns observed for *AtABCC13/MRP11* in *A*. *thaliana* [62] and wheat *TaABCCs* for this hormone [5]. The genes (*RgABCC12*, *RgABCC17* and *RgABCC19*) were highly induced by ETH treatment. The results showed similar *FvABCCs*’ expression in strawberry [2]. Thus, the *RgABCCs* exhibited specific transcription patterns under these hormone treatments, implying their functional diversity in response to these stresses.

Many ABCCs in eukaryotes are involved in the transport of exogenous heavy metals [5,63]. The transcription of *AtABCC6* gene was induced by Cd treatment during seedling development [64]. The transcription of four *TaABCCs* in wheat was activated under Cd exposure [5]. Additionally, two *BnaABCCs* were upregulated under Cd treatment [20]. Here, most *RgABCCs* were induced by Cd treatment, especially *RgABCC1*, which showed the highest expression. RgABCC1 clustered with AtABCC1 and AtABCC2, which are involved in detoxification and tolerance to Cd stress [48], implying that the genes could be specifically involved in Cd transport and Cd stress regulation.

### RgABCC1 might participate in the tolerance to Cd stress

In yeast, YCF1 as an ABCC transporter is a crucial factor and is involved in Cd toxicity tolerance [25,65]. *ΔYCF1* mutant has been classically utilized to confirm the functional role of ABCC transporters from some plants [13,25–27]. Some ABCC/MRPs are important for Cd and other heavy metal transports and have been found in several plants [5,20,30,33]. Here, the significant expression of *RgABCC1* in *R*. *glutinosa* roots was induced by Cd stress, and its complemented expression in the yeast *ΔYCF1* mutant resulted in a Cd tolerant phenotype, suggesting that RgABCC1 might be responsible for Cd transport (Fig 7). Some plant ABCC/MRPs can transport Cd into the vacuole, increasing the tolerance to Cd stress by sequestration [2,13,46,49]. Our data also revealed that RgABCC1 was localized in the vacuole, implying its involvement in the Cd transport into the vacuole and possibly enhancing Cd tolerance. It would be valuable to screen the remaining RgABCCs for the functional rescue of YCF1 sensitivity to Cd stress. Although the molecular function details of these RgABCCs have yet to be verified by further experimentation, our study provides molecular evidence that these transporters might play a general role in heavy metal transport in *R*. *glutinosa*.

## Conclusion

Our study first identified a set of *RgABCC* subfamily genes, examined their spatial-temporal expression patterns, and revealed their differential transcription under various conditions, suggesting their importance in abiotic stress responses. Additionally, functional complementation analysis revealed that RgABCC1 may possess Cd transport activity. The insights provided herein serve as a better understanding of the RgABCC functions in *R*. *glutinosa* that could be used to decipher the transport of diverse specialized metabolites, promote growth and development, and enhance the tolerance to various abiotic stresses.

## Supporting information

S1 Fig The constructs for the *RgABCCs*. (CaMV35S:GFP-RgABCC1, a; CaMV35S:GFP-RgABCC3, b; CaMV35S:GFP-RgABCC11, c; CaMV35S:GFP-RgABCC18, d and PYES2-RgABCC1, e).
(DOCX)

S1 Table Number of the identified ABCCs from some reported plant species. (XLS)

S2 Table The primer sequences of the *RgABCCs* used to clone and construct vectors. The red fonts represent the sequences of these vector adapters.
(XLS)

S3 Table Primer sequences used for the qRT-PCR analysis of the *RgABCC*s. (XLS)

S4 Table Summary of the putative ABCC transporters from the *R*. *glutinosa* transcriptome data. (XLS)

S5 Table Substrates of the functionally characterized ABCCs from other plant species. (XLS)

S6 Table Sequence identities among the RgABCCs. (XLS)

S7 Table Sequence identities of the RgABCCs with other characterized ABCC/MRPs from plant species. (XLS)

S8 Table Relative transcript abundances of the *RgABCCs* in various tissues of *R*. *glutinosa*. (XLS)

S9 Table Relative transcript abundances of the *RgABCCs* in *R*. *glutinosa* roots of different development stages. (XLS)

S10 Table Relative transcript abundances of the *RgABCCs* under various conditions. (XLS)

S11 Table OD_600_ values of cell densities form the different genotype yeasts under CdCl_2_ stress at various concentrations. (XLS)
