# Supplementary material for: Transcriptome-based identification and expression characterization of RgABCC transporters in Rehmannia glutinosa
Source: PLoS One. 2021 Jun 25;16(6):e0253188. doi: 10.1371/journal.pone.0253188 (PMC8232422; doi:10.1371/journal.pone.0253188)

**S1 Fig. The constructs for** **the *RgABCCs*.** (CaMV35S:GFP-RgABCC1, a; CaMV35S:GFP-RgABCC3, b; CaMV35S:GFP-RgABCC11, c; CaMV35S:GFP-RgABCC18, d and PYES2-RgABCC1, e).


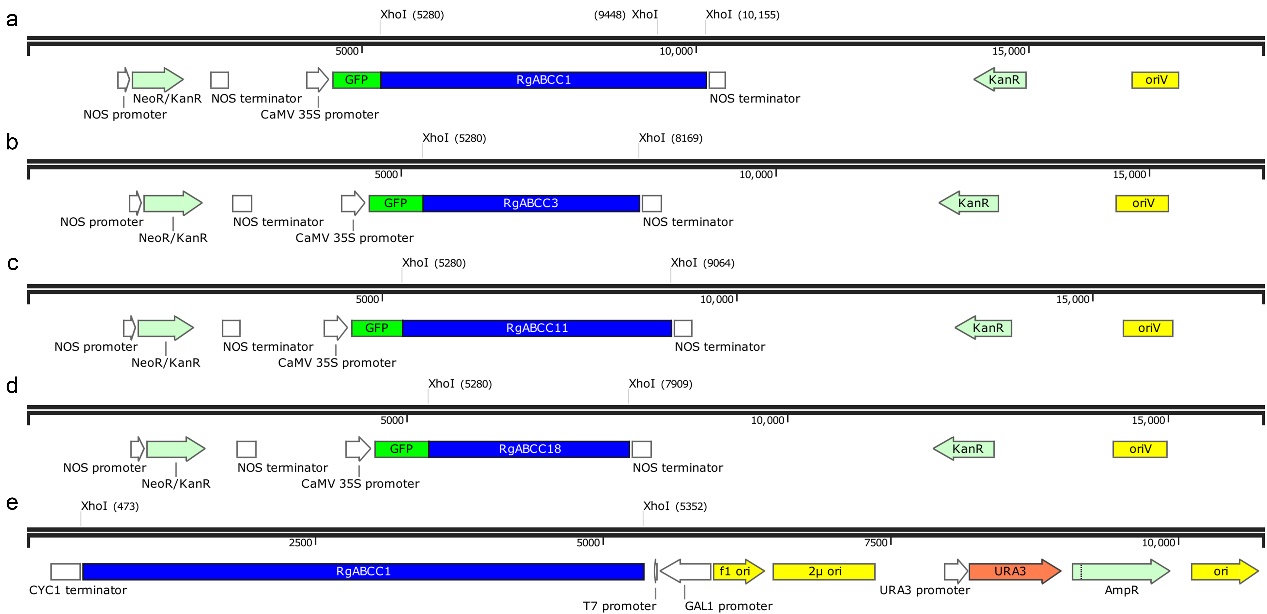

Supplement: S1 Fig — (CaMV35S:GFP-RgABCC1, a; CaMV35S:GFP-RgABCC3, b; CaMV35S:GFP-RgABCC11, c; CaMV35S:GFP-RgABCC18, d and PYES2-RgABCC1, e). (DOCX) [file pone.0253188.s001.docx]
